# Turning immune checkpoint inhibitors into synthetic switches for engineered T cells

**DOI:** 10.1016/j.omton.2026.201346

**Published:** 2026-09-25

**Authors:** Yan-Ruide Li

**Affiliations:** 1 Department of Microbiology, Immunology & Molecular Genetics, University of California, Los Angeles, Los Angeles, CA 90095, USA; 2 Department of Bioengineering, University of California, Los Angeles, Los Angeles, CA 90095, USA

## Main text

Adoptive T cell therapy and immune checkpoint blockade have independently transformed cancer immunotherapy, yet durable responses in solid tumors remain limited by antigen heterogeneity, immunosuppressive tumor microenvironments, and inadequate control over engineered immune cell activity.^1^^,^^2^ Numerous strategies have sought to combine these therapeutic modalities, most commonly by using checkpoint inhibitors to relieve T cell dysfunction or by genetically disrupting inhibitory pathways^3^ (Figure 1). In this issue of Molecular Therapy Oncology, Schaukens and colleagues introduce a conceptually distinct strategy: a mutated PD-1 receptor-based chimeric antigen receptor (PD-1^K78A^ CAR) that retains binding to clinically approved anti-PD-1 antibodies, while eliminating recognition of PD-L1.^4^ In this system, anti-PD-1 antibodies are repurposed from checkpoint-blocking drugs into externally administered molecular switches for engineered T cells (Figure 1).

**Figure 1:**
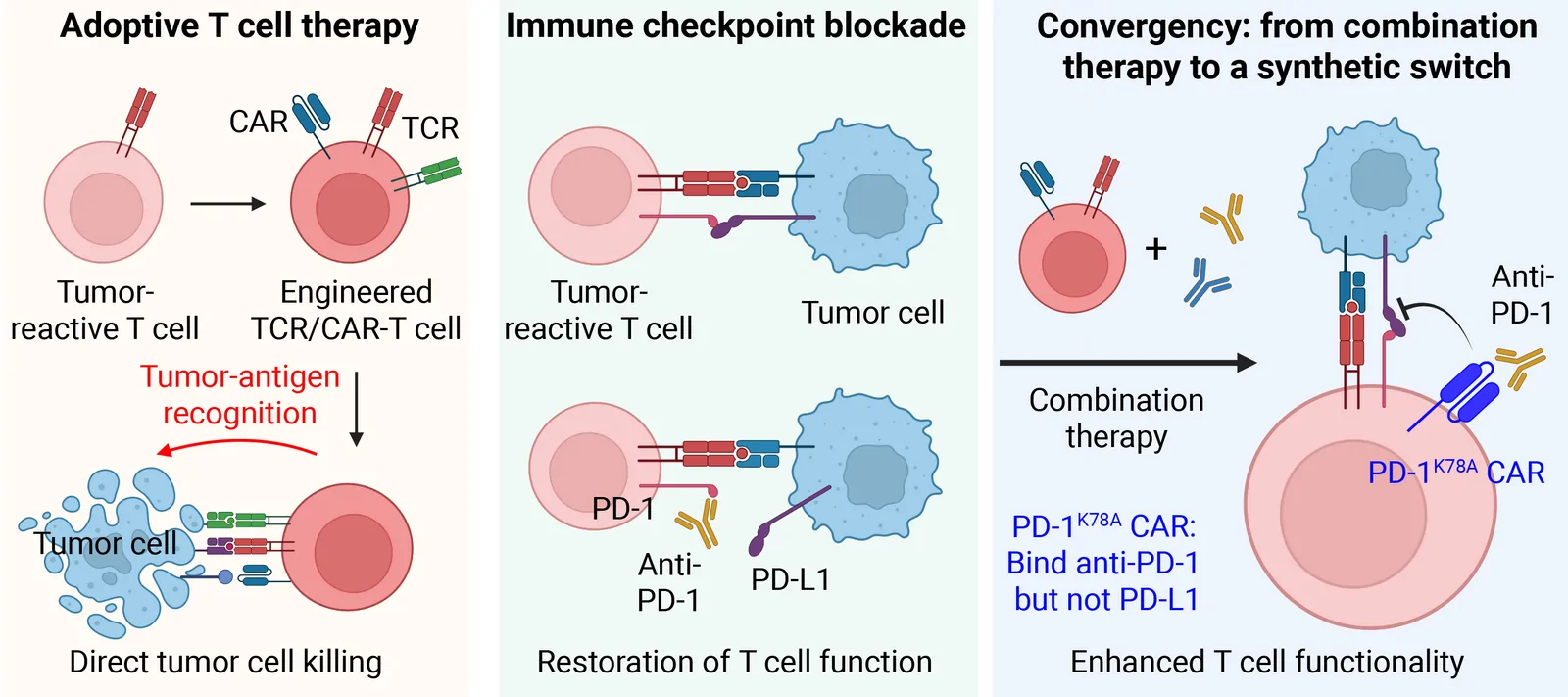
Convergence of adoptive T cell therapy and immune checkpoint blockade toward antibody-controlled engineered T cells Adoptive T cell therapies, including those based on TCR- and CAR-engineered T cells, enhance tumor recognition and cytotoxicity, whereas immune checkpoint blockade with anti-PD-1 antibodies restores T cell function by disrupting inhibitory PD-1/PD-L1 signaling. Conventional combination strategies integrate these approaches to enhance the activity of engineered T cells. A distinct synthetic strategy was developed in which the PD-1^K78A^ CAR is engineered to abolish PD-L1 recognition, while retaining binding to clinically approved anti-PD-1 antibodies. Consequently, anti-PD-1 antibodies can serve as externally administered activating inputs that promote CAR signaling and enhance the activity of tumor-targeting T cells. The figure was generated using BioRender.

The PD-1/PD-L1 pathway is a central regulator of T cell activity and an important mechanism of tumor immune evasion.^5^^,^^6^ Engagement of PD-1 on activated T cells by PD-L1 suppresses proliferation, cytokine production, and cytotoxic function. Antibodies targeting PD-1 or PD-L1 can release this inhibitory brake and have generated durable responses across multiple malignancies, but only a subset of patients derive lasting benefit. CAR T cells offer a complementary strategy by genetically programming T cells to recognize defined cell-surface antigens. Although remarkably effective in several hematologic malignancies, CAR T cell therapy has encountered substantial barriers in solid tumors, including poor trafficking and persistence, immunosuppression, and on-target, off-tumor toxicity caused by antigen expression in normal tissues.^7^

Synthetic biology is increasingly addressing these limitations by moving CAR design beyond simple antigen recognition toward programmable control of immune cell behavior. Switch receptors, logic-gated circuits, and pharmacologically regulated CARs can integrate environmental or externally administered signals to shape T cell activity. PD-1 itself has previously been exploited in this context. For example, PD-1-CD28 switch receptors convert PD-L1 engagement from an inhibitory interaction into a stimulatory signal.^8^ However, using PD-L1 as an activating ligand introduces a potential safety concern because PD-L1 is not tumor specific and can be induced on normal immune and tissue cells, particularly during inflammation.

Schaukens and colleagues invert this paradigm. Rather than engineering T cells to become activated by PD-L1, they make activation dependent on administration of an anti-PD-1 antibody (Figure 1). The authors introduced a K78A substitution into the extracellular domain of PD-1, disrupting its interaction with PD-L1 and PD-L2, while preserving recognition by clinically relevant anti-PD-1 antibodies.^4^ Incorporated into a CAR-containing 4-1BB and CD3ζ signaling domains, PD-1^K78A^ no longer responded to PD-L1-expressing target cells, whereas nivolumab and pembrolizumab retained receptor binding. A single amino acid substitution, therefore, uncoupled the synthetic receptor from its endogenous ligand, while preserving pharmacological accessibility.^4^

This molecular rewiring changes the receptor’s activation logic from dependence on endogenous PD-L1 engagement to pharmacologically controlled activation by an administered anti-PD-1 antibody. When PD-1 receptor-based CARs were transiently expressed by mRNA electroporation, soluble nivolumab or pembrolizumab induced CAR-dependent activation. For PD-1^K78A^, tumor recognition can, therefore, be supplied independently by another tumor-targeting receptor, while anti-PD-1 administration provides an externally controllable activating input. Rather than merely removing inhibitory PD-1 signaling, as occurs during conventional checkpoint blockade, the antibody becomes an activating ligand for a synthetic cellular circuit.^4^

An important mechanistic insight emerged from comparing mRNA- and lentiviral-mediated CAR expression.^9^ Despite retaining antibody binding, lentivirally expressed PD-1 CARs were poorly activated by soluble anti-PD-1 antibodies, whereas the substantially more abundant mRNA-expressed receptors responded efficiently. Receptor expression following mRNA delivery reached markedly higher levels, suggesting that antibody binding alone is insufficient for signaling and that a threshold receptor density is required to enable productive clustering. This observation has implications beyond PD-1 CARs; for synthetic receptors responding to soluble ligands, receptor abundance, geometry, and membrane organization may be as important as ligand affinity or intracellular signaling architecture.

The authors elegantly reinforced this conclusion by manipulating ligand crosslinking.^4^ Immobilized nivolumab restored the activation of lentivirally expressed PD-1 CARs, while increasingly multivalent antibody presentation further enhanced signaling. These findings suggest that receptor density and ligand organization represent interconnected design parameters. They also raise the possibility that biomaterials, nanoparticles, or other multivalent antibody-presenting platforms might eventually provide spatial control over synthetic receptor activation.^10^ Whether such strategies can achieve appropriate biodistribution and safety *in vivo* remains an important question.

Crucially, antibody-responsive CAR signaling could complement, rather than replacing, tumor-specific recognition. Primary T cells were co-engineered with PD-1 receptor-based CARs and an HLA-A2-restricted T cell receptor targeting the melanoma antigen gp100. Nivolumab enhanced tumor-cell killing by T cells carrying PD-1 receptor-based CARs, with significant enhancement observed for PD-1^K78A^ relative to the control CAR condition.^4^ This configuration suggests an appealing two-input architecture in which tumor-specific recognition directs the cellular response, while an externally administered antibody provides an additional pharmacologically controllable activating signal.

The broader significance of this work lies in treating clinically established biologics as inputs for synthetic cellular circuits. Antibodies and engineered cell therapies are generally developed as distinct modalities, even when used in combination. Here, a therapeutic antibody is effectively transformed into an instruction for a genetically engineered immune cell. In principle, this concept could extend beyond PD-1. Natural receptors might be engineered to retain recognition by selected therapeutic antibodies, while eliminating interactions with endogenous ligands and generating orthogonal antibody-receptor pairs capable of controlling activation, co-stimulation, cytokine production, or other cellular programs. Rather than encoding every function permanently within increasingly complex cellular constructs, some behaviors might ultimately be controlled pharmacologically after cell infusion.

Important translational challenges remain. Foremost is the dependence of activation on receptor abundance and crosslinking. High but transient expression following mRNA electroporation enabled activation by soluble antibody, whereas lower stable expression following lentiviral transduction was insufficient under comparable conditions. Increasing stable receptor expression might improve responsiveness but could also increase tonic signaling, differentiation, or exhaustion. Conversely, repeated mRNA administration could preserve transient control but complicated manufacturing and treatment. Defining a therapeutic window for receptor density is, therefore, essential.

It is also unclear whether sufficient antibody-mediated crosslinking will occur *in vivo*. Fc receptor-expressing cells might facilitate multivalent antibody presentation, but this will depend on tissue context, antibody concentration, Fc receptor distribution, and other pharmacological variables. Moreover, the long systemic half-lives of nivolumab and pembrolizumab may sustain activation but could limit rapid reversibility. Quantitative relationships among antibody concentration, receptor occupancy, receptor density, crosslinking, and downstream function will, therefore, need to be established. Of note, the current study remains *in vitro* proof of concept. Future work must determine whether systemic antibody administration can reliably control engineered cells *in vivo*, whether repeated dosing maintains predictable responses, and whether enhanced activity can be achieved without systemic T cell activation or other toxicities. Manufacturing consistency and the potential immunogenicity of the non-native PD-1^K78A^ receptor also require investigation.

The most provocative implication of this study is, therefore, broader than PD-1^K78A^ itself. Synthetic immunology has largely focused on engineering immune cells to sense biological features of tumors. Schaukens and colleagues demonstrate the potential value of engineering cells to sense drugs as well. If receptor specificity, expression, crosslinking, and pharmacology can be appropriately tuned, clinically established antibodies could become programmable molecular inputs for living medicines, which can transform checkpoint inhibitors from passive partners of adoptive cell therapy into externally administered instructions for engineered immune cells.

## Declaration of interests

The author declares no competing interests.
